# Hyperbaric Oxygen Therapy in Burn Care: A Systematic Review of Current Evidence

**DOI:** 10.1093/jbcr/irag026

**Published:** 2026-02-17

**Authors:** Julissa Molina-Vega, Rachel E Pferdehirt, Andrew J Vardanian

**Affiliations:** David Geffen School of Medicine, University of California, Los Angeles, Los Angeles, CA 90095, United States; Division of Plastic and Reconstructive Surgery, University of California Los Angeles, Los Angeles, CA 90095, United States; Division of Plastic and Reconstructive Surgery, University of California Los Angeles, Los Angeles, CA 90095, United States

**Keywords:** hyperbaric oxygen therapy, hyperbaric oxygenation, burn, wound management, wound healing

## Abstract

Hyperbaric oxygen therapy (HBOT) involves inhaling 100% oxygen at pressures exceeding one atmosphere within a chamber. It is used across several specialties and has been proposed as an adjunct in burn management to enhance healing and improve outcomes. Despite increasing interest, evidence supporting its efficacy in burn care remains inconsistent, with no clear consensus in practice. A literature search was conducted in September 2025 across PubMed, Cochrane Library, and Embase without date restrictions to identify studies evaluating HBOT for burn treatment. Inclusion criteria were human studies, English-language articles, and full-text availability. Study selection followed the Preferred Reporting Items for Systematic Reviews and Meta-Analyses (PRISMA) guidelines. Two reviewers screened titles and abstracts, and a third resolved discrepancies. Eligible studies described HBOT as a burn treatment and reported clinical outcomes. Thirteen studies met inclusion criteria: 5 randomized controlled trials, 7 cohort studies, and 1 case–control study, evaluating 566 burn patients. Burn severity, HBOT protocols, and outcomes varied substantially. Several studies reported reduced need for surgery and shorter hospital stays with HBOT. Trends toward improved healing and lower infection risk were noted, while mortality benefits were inconsistent. Heterogeneity in design and treatment regimens limited synthesis via meta-analysis. Hyperbaric oxygen therapy shows promise as an adjunct in burn care, improving healing and reducing complications. However, variability and inconsistent outcomes limit definitive conclusions. Well-designed randomized trials are needed to establish standardized protocols and clarify their clinical role in burn management. Until then, burn centers may consider HBOT for acute injuries and threatened grafts.

## INTRODUCTION

Each year in the United States, over 2 million burn injuries occur, with more than 75 000 requiring hospitalization and approximately 14 000 resulting in death.^1^ Burn injuries are complex and often life-threatening, involving both localized tissue destruction and widespread systemic inflammatory response.

Burn wounds are typically characterized by three distinct zones: (1) a central zone of coagulation, where tissue necrosis and complete capillary occlusion occur, (2) a surrounding zone of stasis, with reduced perfusion and risk of progression, and (3) an outer zone of erythema, which typically recovers unless secondary factors cause additional damage, such as infections.^2^ The central zone can expand significantly within the first 48 hours post-injury, with continued progression of tissue damage for up to 72 hours, often leading to ischemic necrosis. Edema develops rapidly at the injury site due to increased capillary permeability, impaired lymphatic drainage, and altered oncotic pressures. Importantly, edema can also occur in distant, uninjured tissues, owing to the systemic inflammatory response, and ultimately contributing to systemic complications.

Ongoing tissue damage is exacerbated by reduced oxygen delivery, impaired nutrient supply, and decreased perfusion. As such, interventions that may contribute to edema reduction, enhanced oxygenation, improved perfusion, and inflammatory response modulation/mitigation can significantly improve outcomes. To this end, hyperbaric oxygen therapy (HBOT) has been explored as a potential adjunctive treatment in burn care, with the goal of reducing morbidity and mortality while promoting wound healing, improving functional recovery, and minimizing scarring.

Hyperbaric oxygen therapy involves the inhalation of 100% oxygen at elevated atmospheric pressures—typically between 2.0 and 3.0 atmospheres absolute (ATA)—within a hyperbaric chamber. Treatment sessions generally range from 60 to 120 minutes and may be administered one to three times daily, over the course of 5-40 total treatments. No universally accepted protocol currently exists for HBOT administration in burn care.

By significantly increasing oxygen delivery to hypoxic wound tissues, HBOT enhances key processes in wound healing, including angiogenesis, collagen synthesis, and epithelial regeneration. It also exhibits antimicrobial effects, aiding in infection control and reducing the risk of sepsis.^3^ Additionally, HBOT has been associated with reduced edema and inflammation, decreased fluid resuscitation requirements, and lower rates of progression from partial- to full-thickness burns.^4^ HBOT has been shown to alter expression of inflammatory markers, such as reactive oxygen species, nitric oxide, interleukin-6 and -10, which in turn downregulate cell-signaling pathways and inflammatory cascades.^5^

Despite these potential benefits, the incorporation of HBOT into standard burn care protocols remains controversial. Challenges include variability in clinical outcomes, limited access to hyperbaric facilities, and the absence of standardized treatment guidelines for burns. This review aims to examine the role of HBOT in burn management, evaluating its clinical benefits, commonly used treatment protocols, and the current limitations hindering its widespread adoption.

## MATERIALS AND METHODS

### Literature search strategy

A comprehensive literature search was performed in September 2025, with no restriction on publication year. The electronic databases PubMed, Embase, and the Cochrane Library were searched, in accordance with the Preferred Reporting Items for Systematic Reviews and Meta-Analyses (PRISMA) guidelines (Table 1). The search strategy combined keywords related to *“hyperbaric medicine,” “hyperbaric oxygen therapy,”* and *“burns.”* In addition, the reference lists of all included studies were screened manually to identify additional eligible publications not captured by the electronic searches.

**Table 1:** A Table Summarizing Essential Details of Each Study, Including Author, Year of Publication, Percentage of Total Body Surface Area, Total Number of Patients, HBOT Protocols, and Outcomes

**Author and year**	**Study design**	**% TBSA**	**Total patients**	**HBOT protocol**	**Outcomes measured**	**Findings**
Chiang et al. 2017	Retrospective cohort study	<23 to ≥60	53 pts: 38 received HBOT	One daily session for 90 min at 2.5 ATA, 5 days per week	Serum procalcitonin, TBSA of skin graft, number of operations, length of hospitalization	Serum procalcitonin normalized more rapidly in the HBOT group (*P* = .007). No significant differences in TBSA of skin graft (*P* = .587), number of operations (*P* = .710), or length of hospitalization (*P* = .600).
Cianci et al. 1989	Retrospective cohort study	18-39	20 pts: 8 received HBOT	Two daily sessions for 90 min at 2.0 ATA	Length and cost of hospitalization, number of operations	HBOT shortened hospital stay (20.8 vs 33 days; *P* < .012), with fewer surgical procedures (1.3 vs 1.7; *P* = .42), and lower costs ($44 838 vs $55 650; *P* = .47).
Cianci et al. 1990	Retrospective cohort study	19-50	21 pts: 10 received HBOT	Two daily sessions for 90 min at 2.0 ATA	Length and cost of hospitalization, number of operations	HBOT reduced hospital stay by 34% (*P* < .043), surgical procedures by 39% (*P* = .19), and costs by 34% (*P* = .22).
Grossman 1978	Retrospective cohort study	20-90	381 pts: 138 received HBOT	2-3 daily sessions for 7 days, then daily for 90 min at 2.5 ATA	Mortality rate	HBOT reduced mortality rate (41.7% vs 68%).
Jones et al. 2015	Retrospective cohort study	≤7	18 pts: 7 received HBOT	20-30 sessions total	Length of hospitalization	HBOT increased hospital stay (*P* = .0159).
Nygaard et al. 2021	Retrospective cohort study	1-100	13 044 pts: 67 received HBOT	N/A	Length of hospitalization, number of procedures, and mortality rate	HBOT increased mortality rate (29.9% vs 17.5%; *P* = .01). No significant differences in hospital stay (24.6 vs 21.8 days; *P* = .465), or number of procedures (12.5 vs 9.8; *P* = .227).
Chen et al. 2018	Retrospective case–control study	<60	35 pts: 18 received HBOT	One daily session for 120 min at 2.5 ATA, minimum of 20 sessions	Pain score, satisfaction, number of procedures, infection rate, length of hospitalization, scar progression	HBOT showed greater pain score improvement (*P* = .004) and higher satisfaction (*P* = .009). No significant differences in number of procedures, infection, hospital stay, or scar progression.
Özdemir et al. 2023	Prospective cohort study	5-20	58 pts: 29 received HBOT	One daily session for 90 min at 2.4 ATA, 5 days per week, maximum of 21 sessions	Number of surgeries and grafts, epithelization time, satisfaction, infection, length, and cost of hospitalization	HBOT reduced the need for surgery (*P* = .003) and grafting (*P* = .03), accelerated epithelialization (*P* < .001), and improved satisfaction (*P* = .03). Hospital stay (*P* = .169), infection rate (*P* = 1.0), and treatment cost (*P* = .36) were lower, although not statistically significant.
Niezgoda et al. 1997	RCT	<1	12 pts: 6 received HBOT	Two daily sessions for 90 min at 2.4 ATA, 3 days total	Wound hyperemia, size, and exudation	HBOT group showed a 42% reduction in wound hyperemia (*P* = .05), a 35% reduction in the size of wound (*P* = .03), and 22% reduction in wound exudation (*P* = .04).
Waisbren et al. 1982	RCT	About 54	72 pts: 36 received HBOT	N/A	Mortality rate, length of hospitalization, and number of procedures	HBOT was associated with fewer nonsegmented polymorphonuclear leukocytes and fewer grafts, while mortality and hospital stay were similar between groups.
Chong et al. 2013	RCT	≤35	17 pts: 8 received HBOT	Two sessions within 22 hr, for 90 min at 2.4 ATA	WBC count, plasma cytokine inflammatory markers	HBOT showed no significant effect on WBC count or plasma cytokine inflammatory markers compared with control.
Hart et al. 1974	RCT	10-50	191: 138 received HBOT	Three sessions within 24 hr, then 2 sessions, for 90 min at 2.0 ATA	Healing time, fluid requirements, mortality rate	HBOT shortened healing time (19.7 vs 43.8 days, *P* < .005), reduced mortality, and lowered fluid requirements (2.2 vs 3.4 mL/kg/%TBSA)
Brannen et al. 1997	RCT	20-53	125: 63 received HBOT	Two daily sessions for 90 min at 2.0 ATA, minimum of 10 sessions	Length of hospitalization, mortality, number of surgeries	No significant difference in length of hospitalization, mortality, or number of surgeries

Abbreviations: ATA, atmospheres absolute; HBOT, hyperbaric oxygen therapy; TBSA, total body surface area.

### Study selection

Two reviewers independently screened all titles and abstracts for relevance. Full texts of potentially eligible articles were retrieved and assessed against the inclusion criteria. Any disagreements between the two reviewers were resolved through a third reviewer.

### Eligibility criteria

Studies were considered eligible if they met the following criteria: (1) prospective or retrospective design, including cohort studies, case–control studies, case series with more than five participants, or randomized controlled trials; (2) full-text publication in English; (3) human subjects; and (4) evaluation of HBOT in the management of burns. Studies that did not meet these criteria were excluded.

### Data extraction

Data was extracted using a standardized form. Extracted variables included study characteristics (author, year, design), patient population, HBOT protocol (pressure, duration, frequency, total sessions), and reported clinical outcomes.

## RESULTS

**Figure 1:**
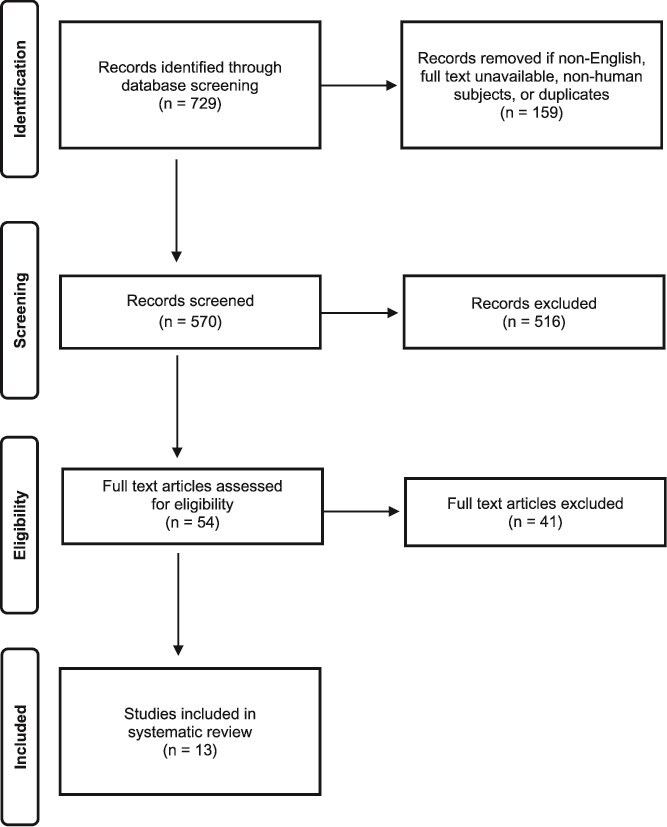
PRISMA Diagram Demonstrating the Number of Records Identified, Screened, Assessed for Eligibility, and Included in the Final Review

The primary search identified 729 articles. After removing duplicates and applying exclusion criteria, 570 articles were eligible for further review. Screening titles and abstracts resulted in 54 articles of potential relevance, which were obtained for full-text review. Ultimately, 13 articles met the inclusion criteria for this study (Figure 1).

Of the 13 included articles, 7 were cohort studies, 1 was a case–control study, and 5 were randomized controlled trials. The included studies were completed from 1974 to 2023. Across all included studies, 566 patients received HBOT, while 13 481 did not. The outcomes measured varied between studies and included length of hospitalization, number of operations, mortality rate, infection rate, and wound parameters, including size, exudation, epithelization, and inflammatory markers. A summary of the included studies is shown in Table 1. The high variability in outcome measures prevented the performance of a meaningful meta-analysis. Additionally, the HBOT protocols differed considerably in number of sessions, frequency, and pressure (Table 2). Lastly, substantial variability in reported outcomes was observed across studies, in part due to non-equivalent patient populations, as evidenced by wide discrepancies in total body surface area (TBSA) burned.

**Table 2:** A Table Summarizing HBOT Protocol Parameters across Studies, Including Total Number and Frequency of Sessions, Session Duration, and Treatment Pressure

**Author and year**	**HBOT frequency**	**Duration (min)**	**Pressure (ATA)**	**Total sessions**
Chiang et al. 2017	Once daily	90	2.5	N/A
Cianci et al. 1989	Twice daily	90	2.0	N/A
Cianci et al. 1990	Twice daily	90	2.0	N/A
Grossman 1978	2-3 sessions daily for first 7 days, then once daily	90 (or 45 for children)	2.5 (or 2.0 for children)	N/A
Jones et al. 2015	N/A	N/A	2.0	10-20, up to 30 if surgery required
Nygaard et al. 2021	N/A	N/A	N/A	N/A
Chen et al. 2018	Once daily	120	2.5	Minimum 20
Özdemir et al. 2023	Once daily	90	2.4	Maximum 21
Niezgoda et al. 1997	Twice daily	90	2.4	6
Waisbren et al. 1982	N/A	N/A	N/A	N/A
Chong et al. 2013	Two sessions within 22 hr	90	2.4	2
Hart et al. 1974	Three sessions within 24 hr, then twice daily	90	2.0	N/A
Brannen et al. 1997	Twice daily	90	2.0	Minimum 10

Abbreviation: HBOT, hyperbaric oxygen therapy.

## DISCUSSION

Burns remain a major cause of morbidity and mortality, with outcomes largely determined by burn characteristics, depth, TBSA, and time to intervention. Burn depth is influenced by both the intensity and duration of exposure. The initial burn injury is characterized by a central zone of coagulation necrosis, surrounded by zones of stasis and hyperemia. However, persistent ischemia, which may result from delayed intervention, can lead to propagation of burn injury and greater TBSA involvement. For this reason, optimized early intervention is critical.

Ischemia develops rapidly due to impaired tissue perfusion, while edema arises through multiple mechanisms, including decreased oncotic pressure, increased capillary permeability, and altered interstitial space compliance.^6^ Early surgical debridement, skin grafting, wound closure, and appropriate antibiotic therapy are critical to minimize ischemia, prevent infection, and reduce the risk of sepsis. Fluid resuscitation is equally important to restore circulating volume, maintain organ perfusion, and prevent multi-organ dysfunction.^3^

### Wound healing and infection control

Hyperbaric oxygen therapy has been investigated as a potential adjunctive therapy to optimize healing, preserve tissue viability, improve blood flow, and reduce complications.^7^ Hyperbaric oxygen therapy has been shown to attenuate leukocyte adhesion, thereby reducing subsequent inflammatory damage.^8^ Additionally, it exerts anti-inflammatory effects by alleviating tissue hypoxia and downregulating key inflammatory mediators.^8^ HBOT may improve wound healing and infection control by reducing edema, enhancing circulation through vasoconstriction, and limiting burn wound progression.^9^ Hart et al. conducted a randomized controlled trial stratifying patients by percentage of the TBSA. They reported significantly faster wound healing in the HBOT group compared with controls (19.7 vs 43.8 days, *P* < .005), along with reduced fluid requirements, and a lower mortality rate.^10^ However, clinical outcomes remain mixed. Following a mass casualty incident in Taiwan, Chiang et al. found no significance differences in skin graft requirements or hospital stay between HBOT and control groups. They did note that HBOT was associated with faster normalization of serum procalcitonin levels (a marker of sepsis), suggesting reduced sepsis risk.^3^ In contrast, Waisbren et al. reported higher rates of sepsis and renal complications in the HBOT group, despite a lower percentage of nonsegmented polymorphonuclear leukocytes, indicating a reduced acute inflammatory response.^11^ Similarly, Chong et al. found no significant differences in cytokine markers or white blood cell counts but observed fewer positive tissue cultures in the HBOT group, suggesting a potential antimicrobial effect.^12^ In controlled burn wounds inflicted on healthy volunteers, Niezgoda et al. reported a 42% reduction in wound hyperemia, 35% reduction in wound size, and 22% reduction in wound exudate, but no improvement in re-epithelization rate, likely due to the rapid natural healing of experimental burns.^13^ Conversely, Özdemir et al. demonstrated significantly faster wound epithelization with HBOT (13.38 days) compared to controls (22.1 days).^14^

### Surgical needs and hospitalization

The impact of HBOT on need for surgical procedures and duration of hospitalization has also been widely studied. Cianci et al. reported that burn patients treated with HBOT had shorter hospital stays and required fewer surgical procedures for debridement and grafting, attributing these findings to reduced edema and limited progression of partial-thickness to full-thickness burns.^4^^,^^15^ Similarly, Özdemir et al. observed lower surgical requirements in the HBOT group, with 10.3% of patients requiring surgery compared to 48.3% in the control group. They also observed reduced grafting rates in partial-thickness burns (3.4% vs 24.1%, respectively) and shorter hospital stays, by an average of 2.65 days.^14^ In contrast, Jones et al. examined patients with diabetic foot burns and found longer hospital stays among those treated with HBOT compared with controls.^16^ Chen et al. likewise reported no significant difference in hospitalization duration between groups and no difference in debridement times.^17^ Nygaard et al. also found no difference in total hospital days; however, they noted that earlier mortality shortened hospital stay in the HBOT group. Importantly, their analysis was limited by major sample size imbalance, with only 67 patients undergoing HBOT compared with 12 977 controls.^7^ Brannen et al. found no difference in hospital stay or number of surgeries in burn patients treated with HBOT compared to patients not treated with HBOT.^18^

### Pain and patient satisfaction

Pain control and patient satisfaction have also been examined. Chen et al. reported improved post-burn pain control and higher patient satisfaction with HBOT, findings that were further supported by Özdemir et al., who also reported greater satisfaction among HBOT-treated patients.^14^^,^^17^

### Mortality and morbidity

Evidence regarding the effects of HBOT on mortality in burn patients remains inconsistent. Grossman et al. reported significantly reduced mortality among patients treated with HBOT compared to controls, whereas Nygaard et al. observed higher mortality in the HBOT group (29.9% vs 17.5%), potentially influenced by confounding factors such as the older age distribution in the HBOT group.^7^^,^^19^ Hart et al. demonstrated a substantial reduction in mortality, reporting improvements of 21%-30% among patients who received HBOT.^10^ Other studies, including those by Waisbren et al., Chiang et al., and Brannen et al., found no significant differences in mortality between HBOT and control groups.^3^^,^^11^^,^^18^ These conflicting findings highlight the need for larger, well-controlled studies to clarify the impact of HBOT on survival outcomes in burn care.

### Cost effectiveness

Finally, the economic implications of HBOT have been considered. Although HBOT is costly, particularly when not covered by insurance, several studies suggest it may reduce overall treatment costs by shortening hospital stays and decreasing the need for surgical interventions. Cianci et al. reported a 19% reduction in total treatment costs among patients receiving HBOT, and in a subsequent study, demonstrated an average savings of $31 600 per patient, corresponding to a 34% reduction.^4^^,^^15^ Similarly, Özdemir et al. found lower costs in the HBOT group compared to controls ($464.52 vs $540.57).^14^ These findings suggest that, despite the upfront costs, HBOT may provide substantial economic benefits in the treatment of burn injuries, though further high-quality evidence is required to confirm this.

### Hyperbaric oxygen therapy protocol

Hyperbaric oxygen therapy protocols demonstrated substantial variability across the included studies in terms of pressure, duration, frequency, and total number of treatments. Treatment pressures ranged from 2.0 to 2.5 ATA, while session frequency varied from one to three times daily. Niezgoda et al. administered 2 sessions for a total of 3 days, whereas Chong et al. delivered two sessions total within 22 hours of admission.^12^^,^^13^ Hart et al. provided three sessions in the first 24 hours, followed by twice daily until wound healing, while Grossman et al. adjusted frequency to two to three sessions daily depending on the percentage of TBSA affected.^10^^,^^19^ Most of the remaining studies employed once or twice daily regimens with a greater total number of sessions. Session length was consistently 90 minutes across studies, except for Chen et al., who used 120-minute sessions.

Although HBOT is generally considered safe, several adverse effects have been reported. Most are mild, self-limiting, and without long-term sequelae. Chiang et al. reported two patients who experienced chest tightness during their initial HBOT session, while Hart et al. described four patients whose therapy was discontinued due to claustrophobia.^3^^,^^10^ Patients with underlying anxiety and claustrophobia require caution, and sometimes sedatives may be used to facilitate treatment. Additional risks of HBOT include barotrauma and tympanic membrane rupture, as well as rare but serious complications such as seizures.^20^ Seizures are typically associated with oxygen toxicity, which can often be avoided by using shorter dive durations or lower pressures. Furthermore, HBOT centers can collaborate with Ear, Nose, and Throat (ENT) specialists to place prophylactical ear tubes when appropriate, minimizing the risk of ear-related complications. Ocular effects, including transient myopia, cataracts, and scotomas, have been described with prolonged therapy, but may resolve after treatment is discontinued.^17^ HBOT may also cause transient hypoglycemia and oxygen toxicity affecting pulmonary, cardiology, and neurological systems, particularly in patients with preexisting airway obstruction or congestive heart disease, which increases the risk of pulmonary barotrauma, air trapping, and pulmonary edema.

Beyond safety considerations, access to HBOT is constrained by the limited availability of hyperbaric chambers in burn centers and specialized clinics.^7^ Furthermore, some patients may be too critically ill following severe burns to tolerate prolonged HBOT sessions; however, this risk can be mitigated by using shorter dive durations and ensuring nurse supervision during treatment sessions. Cost and insurance coverage represent additional barriers. Although the Undersea and Hyperbaric Medical Society (UHMS) lists thermal burns among its approved indications, reimbursement varies considerably by insurer, location, severity of injury, and whether standard therapies have failed. As such, safety, access, and cost must be carefully weighted when considering HBOT in burn management.

### Study limitations

There are some limitations in this study. First, the substantial heterogeneity in outcome measures across the included studies limited our ability to perform quantitative analysis. Therefore, outcomes could only be qualitatively summarized. Additionally, publication bias and the restriction to English-language studies may have reduced the number of eligible articles and influenced the overall findings. Lastly, many studies had small sample sizes and unequal distribution of patients between HBOT and control groups, introducing potential selection bias.

## CONCLUSION

This systematic review synthesizes current evidence on the role of HBOT as an adjunctive treatment in burn management. Hyperbaric oxygen therapy has shown promising effects in burn wound management, including enhanced wound healing, improved infection control, reduced progression from partial- to full-thickness burns, and potential decreases in surgical needs and length of hospitalization. However, its overall impact on clinical outcomes remains inconclusive when compared with current standard treatment. Significant variability in study design, HBOT protocols, and patient selection contributed to inconsistent findings regarding mortality, morbidity, and cost-effectiveness. Although existing evidence supports HBOT as a potentially valuable adjunct to standard burn care, high heterogeneity among existing studies limits definite conclusions and generalization. Future large-scale, randomized controlled trials employing standardized HBOT protocols are essential to clarify its efficacy, optimize clinical outcomes, and guide data-driven improvements in burn care. Until such evidence is available, burn centers should consider HBOT as a complementary therapy for selected patients with acute burn injuries, balancing its potential benefits against cost, availability, and individual patient factors.

## Notes

Author contributions: Julissa Molina-Vega (Conceptualization [equal], Data curation [lead], Formal analysis [lead], Investigation [lead], Methodology [lead], Resources [lead], Visualization [lead], Writing—original draft [lead]), Rachel E. Pferdehirt (Supervision [equal], Writing—review & editing [equal]), Andrew J. Vardanian (Conceptualization [equal], Supervision [equal]).

Funding: The authors have no financial interests, including products, devices, or drugs associated with this manuscript. There are no commercial associations that might pose or create a conflict of interest with information presented in this manuscript, such as consultancies, stock ownership, or patent licensing. No funding was used for this manuscript.
